# The effects of botulinum toxin type A injection on pain symptoms, quality of life, and sleep quality of patients with diabetic neuropathy: A randomized double-blind clinical trial

**Published:** 2019-07-06

**Authors:** Hossein Salehi, Moein Moussaei, Zahra Kamiab, Alireza Vakilian

**Affiliations:** 1 Department of Plastic and Reconstructive Surgery, School of Medicine, Rafsanjan University of Medical Sciences, Rafsanjan, Iran; 2 Clinical Research Development Center, Ali Ibn Abi Talib Hospital, Rafsanjan University of Medical Sciences, Rafsanjan, Iran; 3 School of Medicine, Rafsanjan University of Medical Sciences, Rafsanjan, Iran; 4 Department of Social Medicine, School of Medicine, Rafsanjan University of Medical Sciences, Rafsanjan, Iran; 5 Clinical Research Development Unit, Ali Ibn Abi Talib Hospital, Rafsanjan University of Medical Sciences, Rafsanjan, Iran; 6 Department of Neurology, School of Medicine, Rafsanjan University of Medical Sciences, Rafsanjan, Iran; 7 Non-Communicable Diseases Research Center, Ali Ibn Abi Talib Hospital, Rafsanjan University of Medical Sciences, Rafsanjan, Iran

**Keywords:** Diabetic Neuropathies, Botulinum A Toxin, Quality of Life, Sleep

## Abstract

**Background:** Neuropathic pain is one of the most common problems in patients with diabetes mellitus (DM). In this study, the effect of botulinum toxin type A (BTX-A) on neuropathic pain, quality of sleep, and quality of life of diabetic patients with sensorimotor polyneuropathy was studied.

**Methods:** This randomized placebo-controlled trial study was carried out in a double-blind (patient-researcher) method. The study was performed on 32 patients with type 2 DM. Neuropathy was confirmed by Douleur Neuropathique 4 (DN4) Questionnaire and nerve conduction study (NCS). The patients were randomly assigned to two intervention and control groups based on the random numbers table. After selecting the subjects, we used 36-Item Short Form Health Survey (SF-36), Neuropathic Pain Scale (NPS), Visual Analogue Scale (VAS), and Pittsburgh Sleep Quality Index (PSQI) questionnaires before and after 3 months of 100 units BTX-A injection (as intervention group) or same amount of chloride sodium (as control group) to the subjects' feet. The data were analyzed by SPSS software using independent two-sample t-test, chi-square test, and one-way repeated measures analysis of variance (ANOVA).

**Results:** 12 male and 20 female patients participated in this study. There was a significant difference in the mean VAS, PSQI, physical dimension of the SF-36, and some NPS indices over time (12 weeks) (P < 0.001).

**Conclusion:** The results of this study showed that BTX-A reduced neuropathic pain and improved the quality of life and sleep in people with diabetic neuropathy.

## Introduction

Neuropathic pain is one of the most prevalent problems associated with diabetics.^1^ According to the International Association for the Study of Pain (IASP), there are two general types of pain. In the majority of cases, the term ‘pain’ refers to tissue damage caused by external stimuli (nociception). The other category, referred to as ‘neuropathic pain’, occurs due to damage to the peripheral nervous system (PNS) and the central nervous system (CNS), or because of the disturbances in signal transduction along the nerves.^2^^-^^4^ These types of pain attack extremities, thereby disrupting sleep, reducing the quality of life, creating anxiety, interfering with routine activities, and increasing living costs.^5^^-^^7^

Diabetic sensorimotor polyneuropathy (DSPN) is the most prevalent cause of peripheral neuropathy. This condition is developed in 30%-90% of diabetic patients.^7^^,^^8^ Pain, the numbness of organs, and sensory impairment, especially in the lower limbs with a stocking-glove distribution pattern developing towards proximal parts, are the main symptoms of the disease.^1^^,^^9^^,^^10^

The prevalence of DSPN has been reported to be different in various studies, depending on the related diagnostic criteria. Approximately 20 to 30 million people around the world suffer from this condition.^11^^,^^12^ In Iran, the prevalence of this disorder has been reported to be 28% in Khoramabad, 45.6% in Hamadan, and 51.7% in Yazd.^13^^-^^15^

Finding efficient treatments for this disease has always been a serious challenge, with about 39% of the patients with DSPN reported to have been unresponsive.^16^ Drugs, such as carbamazepine, opioids, and antidepressants were developed and used to reduce the rate and extent of neuropathic pain; however, due to the non-long-term pain relief, increased treatment costs, and side effects of these drugs, they have turned into challenging issues.^17^^,^^18^

Neuropathic pain has recently been treated with lidocaine, botulinum toxin type A (BTX-A), and capsaicin. Although the effects of these drugs have not so far been confirmed, they are likely to be used, given the limited number of side effects they may generate in patients.^19^

BTX affects both motor neurons and all other nerves.^20^ Research has also shown that BTX-A’s analgesic effects are independent of reducing muscle disorders, and the dose needed to relieve pain is less than the one necessary to improve the movement.^21^ Paterson et al. found out that BTX-A could block pain receptors; in addition, they observed that the intra-cutaneous injection of BTX-A reduced the sensitivity of mechanical pain and inhibited pain.^22^ In the same vein, other studies reported that BTX-A reduced neurogenic inflammation.^23^

BTX-A is injected in subcutaneous, intramuscular, and muscular forms as well as in the nerves.^24^

Nam et al. reported that the severity of neuropathic pain decreased significantly in a patient aged 62 years with brain tumors after the injection of a 100-unit dose of BTX; the resulted pain relief lasted for 12 weeks.^25^

Park and Park investigated the impact of BTX on neuropathic pain; they referred to the 40-year use of BTX in medicine and reported that treatment with BTX was effective in patients with diabetes mellitus (DM), trigeminal neuralgia (TNG), herpes, as well as intolerable types of neuropathic pain, such as the pain caused by spinal cord injuries as well as post-stroke pain.^26^

Han et al. studied the effects of BTX-A on the treatment of 40 patients with neuropathic pain due to spinal cord injuries. Their study which was a randomized, double blind, and placebo-controlled study showed that BTX-A reduced neuropathic pain caused by spinal cord injuries.^27^

The present study was conducted to compare the effects of BTX-A on lower limb neuropathic pain among two groups of patients with DM, one as an intervention group and the other as control group receiving a placebo, who referred to the Diabetes Clinic of Ali ibn Abi Talib Hospital in Rafsanjan, Iran.

## Materials and Methods

**Figure 1:**
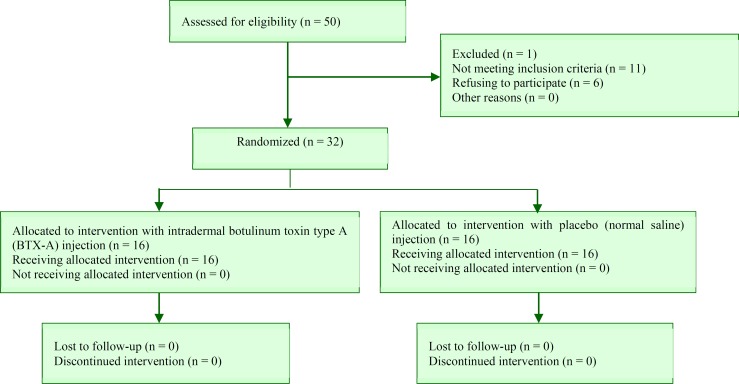
Consort flow diagram of clinical trial

The present study was a double-blind clinical trial with the design shown in the flowchart (Figure 1).

In order to meet ethical criteria, while obtaining the code of ethics from the Ethics Committee (IR.RUMS.REC.1395.116), written informed consent was obtained from the participants. This trial was registered in the Iranian Registry of Clinical Trials (IRCT) system under code of IRCT20190129042540N1.

***Patients:*** Firstly, 50 patients with sensory and motor complaints were evaluated. In fact, 38 patients with type 2 DM aged 40-70 years, who were admitted to the Diabetes Clinic of Ali Ibn Abi Talib Hospital in Rafsanjan, were selected based on the inclusion and exclusion criteria to be listed in the following sections.

The neuropathy (sensory and motor polyneuropathy) of the patients was confirmed by a neurologist using Douleur Neuropathique 4 (DN4) Questionnaire and nerve conduction study (NCS).

***Inclusion criteria:*** The main inclusion criteria included having type 2 DM for at least 3 years, aging 40-70 years, giving positive answers to four or more questions in DN4,^28^^,^^29^ and showing the evidence of diabetic neuropathy in the lower limb electrophysiological examination,^30^ as well as suffering from mild to severe neuropathy according to the Toronto Clinical Neuropathy Score (TCNS).^31^

***Exclusion criteria:*** Initially, subjects allergic to BTX-A, those who used other drugs to reduce neuropathic pain, the ones with a history of myasthenia gravis (MG), subjects with the distal muscle weakness, kidney dysfunctions, and a history of alcohol use, post-operative patients, those who started taking medicines after the beginning of the study, and the ones who developed aminoglycosides or the obstructive airway disease concurrently, were excluded from the study.^32^

**Figure 2:**
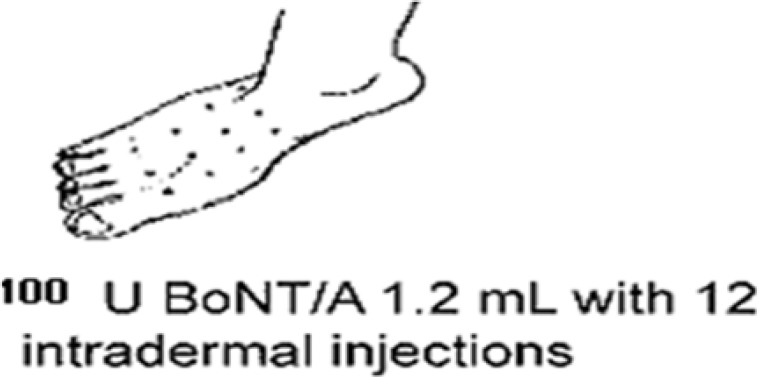
Foot injection method of botulinum toxin type A (BTX-A)

***Method of injection:*** All patients were asked to attend the Diabetes Clinic of Ali ibn Abi Talib Hospital on a specific day. All preparations for the injection of the toxin were made by an expert on the same day and at the same location in the hospital, with necessary measures adopted concurrently by other colleagues. For the patients in the intervention group, 100 units of BTX-A (Dysport, Ipsen, UK) were dissolved in 1.2 ml of normal saline and according to the grid pattern of 12 points (3 × 4) on the foot surface (Figure 2), were injected into the subjects’ leg intradermally, using a 31-gauge 0.5 inch needle as 0.1 ml (8.33 units) injection per each site. Patients in the control group received normal saline at the same amount and with the same pattern.^28^ It is worth noting that before the injection, the patient’s leg was covered with analgesic gels. Given that the symptoms of the relief caused by BTX starts within 5 to 7 days after the injection, the peak time is 14 to 28 days, and the duration of the effect lasts for 3 to 7 months,^33^ the patients were examined within three months after the injection to assess the extent of the treatment and recovery. 

To collect data required in this study, the following tools were used:

***36-item Short Form Health Survey Questionnaire (SF-36):*** The SF-36 standard questionnaire is used to measure the health-related quality of life. Montazeri et al. confirmed, using Cronbach’s alpha, the reliability and validity of the Persian version of the SF-36 standard questionnaire at 0.65. In this questionnaire, 8 physical activity scales, including the variables of physical role functioning, physical pain, general health, vitality, physical functioning, social role functioning, emotional role functioning, and mental health are assessed. The first five scales are used in assessing the physical condition, with the three other scales used to examine mental status.^34^

***Visual Analogue Scale (VAS):*** It includes a questionnaire in which a straight line is drawn, being used to compare the patient’s pain scores at various stages. This questionnaire has been used in numerous studies, with its validity and reliability having been confirmed by Cronbach’s alpha at 0.99.^35^^,^^36^

***Neuropathic Pain Scale (NPS):*** This questionnaire assesses the feeling and quality of pain in patients. It consists of 10 questions, with two questions being related to the severity of pain in general, and 8 questions being connected with the quality and location of pain, including hot sensation, dull sensation, cold sensation, unpleasant sensation, deep sensation, surface sensation, and pain intensity. The validity and reliability of the NPS questionnaire have been confirmed by Jensen et al. using Cronbach’s alpha at 0.78.^37^

***Pittsburgh Sleep Quality Index (PSQI):*** PSQI is a questionnaire for the assessment of the sleep quality of people, being composed of 18 questions, with a scoring scale of 0 to 3. In this questionnaire, 7 subscales of sleep quality, sleep latency, sleep time, sleep efficiency, sleep disorders, sleep deprivation, and daily functional disorders are considered. The scores of these seven components constitute the total score of the questionnaire, ranging from 0 to 21; the higher the score is, the lower an individual’s sleep quality will be. A score over 6 alludes to an undesirable sleep quality. The validity and reliability of this questionnaire was confirmed by Farrahi et al. using Cronbach’s alpha at 0.77.^38^

The independent two-sample t-test and the chi-square test were used to compare demographic and primary features of the patients. In addition, one-way repeated measures analysis of variance (ANOVA) was used to assess the changes to the outcome indices in the intervention and placebo groups in 1, 4, 8, and 12 weeks after the intervention. The data were analyzed using SPSS software (version 20, IBM Corporation, Armonk, NY, USA). The significance level was considered at 0.05. 

The patients were assigned randomly to two intervention and control groups based on random numbers and completed SF-36, NPS, VAS, and PSQI questionnaires. 

The patients were referred back to the clinic one week later and were required to return again in weeks 4, 8, and 12 after the injection to collect the data required for NPS, VAS, SF-36, and PSQI questionnaires, which were used at the beginning of the injection (Figure 2).

## Results

According to the inclusion and exclusion criteria, 32 patients (12 men and 20 women) were divided into two intervention and placebo groups in the present study, with 16 subjects in each group.

According to the results of table 1, the two groups were not significantly different in terms of the type of treatment, sex, mean age, DM duration, and hemoglobin A1c (HbA1c) levels at the beginning of the study, so they were homogeneous (Table 1).

**Table 1:** Frequency distribution of gender and type of treatment and mean and standard deviation (SD) of other underlying variables

**Variable**		**Intervention group (n = 16)**	**Placebo group (n = 16)**	**P**
Type of treatment [n (%)]	Oral	9 (56.25)	9 (56.25)	> 0.999*
	Injection	7 (43.75)	7 (43.75)	
Gender [n (%)]	Male	6 (37.50)	6 (37.50)	> 0.999*
	Female	10 (62.50)	10 (62.50)	
Age (year) (mean ± SD)		5.3 ± 58.3	7.5± 56.7	0.485**
Duration of DM (year)		4.8 ± 15.4	5.8 ± 14.7	0.693**
HbA1C (mean ± SD)		0.8 ± 8.9	1.1 ± 8.6	0.394**

DM: Diabetes mellitus; HbA1C: Hemoglobin A1c; SD: Standard deviation

* Chi-square test,

** T-test

**Table 2:** The rate of changes in mean of pain index variables, in terms of time, time/group, and groups

**Variable/test**		**df**	**F**	**Mean of squares**	**P**
VAS/Greenhouse-Geisser	Time	2.478	57.851	33.816	< 0**.**001
	Time/group	2.478	49.134	28.721	< 0**.**001
	Between groups	1.000	25.344	126.025	< 0**.**001

VAS: Visual analogue scale; df: Degree of freedom

One-way repeated measures ANOVA was used to compare the mean changes in the outcome variables in the two groups over time. In all cases, due to the significance of the Mauchly’s Test of Sphericity, the results of the non-parametric test for Greenhouse-Geisser correction were reported. The only case where the spherical assumption was confirmed using the Mauchly probe (P > 0.050) and sphericity-assumed test results, was the cold sensation variable.

**Figure 3:**
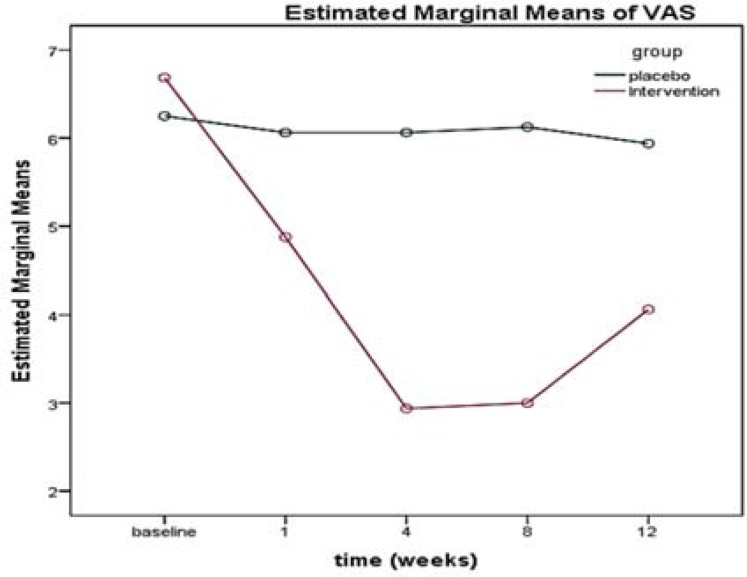
Trend of visual analogue scale (VAS) score in the intervention and control groups over time

There was no significant difference in the VAS between the two groups (P = 0.270) at the beginning of the study, and the two groups were homogeneous. There was a significant difference in the mean VAS over time (12 weeks) (P < 0.001); in addition, there was a significant correlation between the mean VAS in the intervention and control groups, which resulted in a change in the mean VAS. The time was not the same in the two groups, and the decrease in pain intensity was significantly higher in the intervention group than the placebo group during the follow-up period (from the beginning to the end of week 12) (Table 2, Figure 3).

**Figure 4:**
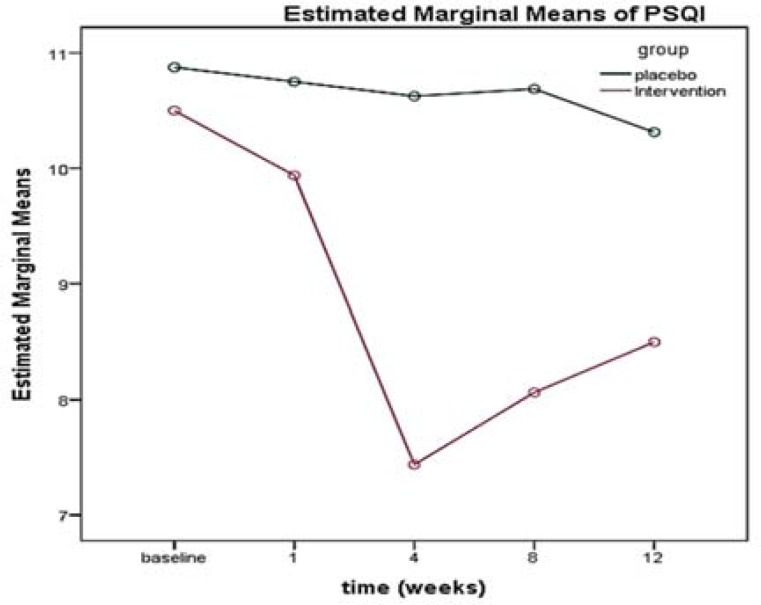
Trend of Pittsburgh sleep quality index (PSQI) score in the intervention and control groups over time

PSQI measured at different times for the two intervention and control groups showed that there was no significant difference between the two groups at the beginning of the study in terms of this specific criterion (P = 0.678). A significant difference was discovered in the mean PSQI variation over time (12 weeks) (P < 0.001); besides, the assessment of the interaction of the PSQI index over time in both groups showed that the drop in the sleep quality score of the subjects in the intervention group was significantly higher than that of the placebo group over time (from the beginning to the end of week 12) (Table 3, Figure 4).

The lower the score of the questionnaire was, the higher the quality of sleep would be.

In addition, there was no significant difference between the two groups in terms of the mean score of the psychological dimension of the quality of life (P < 0.050). Regarding the physical aspect of the quality of life, there was no significant difference between the two groups at the beginning of the study (P = 0.678). However, a significant difference was identified in the mean changes of the physical dimension of the quality of life in the questionnaire over time (12 weeks) (P < 0.001); therefore, our results showed a significantly higher and more noticeable progression of the quality of life's physical dimension in the interventional group compared to the subjects of the placebo group (Table 4).

**Table 3:** The rate of changes in mean of sleep quality questionnaire score by time, time/group, and groups

**Variable/test**		**df**	**F**	**Mean of squares**	**P**
PSQI/Greenhouse-Geisser	Time	2.672	21.824	24.011	< 0.001
	Time/group	2.672	15.251	16.779	< 0.001
	Between groups	1.000	4.021	124.256	0.050
SF-36/Greenhouse-Geisser	Time	1.676	10.077	163.046	< 0.001
	Time/group	1.676	1.428	23.105	0.249
	Between groups	1.000	8.254	945.756	0.007

PSQI: Pittsburgh sleep quality index; SF-36: 36-item short form health survey; df: Degree of freedom

**Table 4:** The rate of changes in mean of neuropathic pain scale (NPS) questionnaire indexes by time, time/group, and groups

**Variable/test**		**df**	**F**	**Mean of squares**	**P**
Sharp sensation/Greenhouse-Geisser	Time	2.741	20.538	8.185	< 0**.**001
	Time/group	2.741	15.366	6.124	< 0**.**001
	Between groups	1.000	2.700	17.556	0.111
Hot sensation/Greenhouse-Geisser	Time	2.746	59.411	22.498	< 0**.**001
	Time/group	2.746	34.435	13.435	< 0**.**001
	Between groups	1.000	16.846	75.625	< 0**.**001
Dull sensation/Greenhouse-Geisser	Time	3.068	25.000	8.394	< 0**.**001
	Time/group	3.068	11.796	3.961	< 0**.**001
	Between groups	1.000	6.987	31.506	0.013
Cold sensation/Sphericity Assumed	Time	4.000	15.298	4.009	< 0**.**001
	Time/group	4.000	10.791	2.828	< 0**.**001
	Between groups	1.000	3.856	15.625	0.050
Sensitive sensation/Greenhouse Geisser	Time	2.687	23.078	10.406	< 0**.**001
	Time/group	2.687	11.627	5.242	< 0**.**001
	Between groups	1.000	3.538	13.225	0.070
Unpleasant sensation/Greenhouse Geisser	Time	3.156	34.944	19.735	< 0**.**001
	Time/group	3.156	17.861	10.087	< 0**.**001
	Between groups	1.000	13.601	44.100	< 0**.**001
Deep sensation/Greenhouse-Geisser	Time	2.279	21.532	20.485	< 0**.**001
	Time/group	2.279	9.898	9.417	< 0**.**001
	Between groups	1.000	3.102	15.006	0.088
Surface sensation/Greenhouse Geisser	Time	2.088	22.038	22.256	< 0**.**001
	Time/group	2.088	8.674	8.760	< 0**.**001
	Between groups	1.000	5.728	28.056	0.023
Pain intensity/Greenhouse-Geisser	Time	2.880	45.639	18.608	< 0**.**001
	Time/group	2.880	22.814	9.302	< 0**.**001
	Between groups	1.000	14.211	65.025	< 0**.**001

df: Degree of freedom

**Figure 5:**
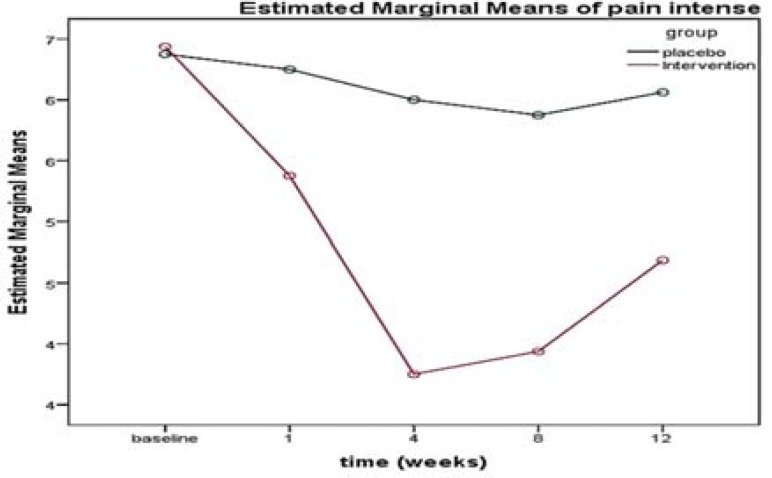
Trend of pain intensity score in the intervention and control groups over time

The results of the NPS questionnaire (sharp sensation, hot sensation, dull sensation, cold sensation, sensitive sensation, unpleasant sensation, surface sensation, pain intensity sensation) are summarized in table 4. According to the results of the present study, there was no significant difference between the two groups in terms of any indices (P < 0.050) (the two groups were the same). The analysis of results of the interactive effects of these indices showed that the score slope of all these indices (except sharp sensation, sensory sensation, and deep sensation) was significantly different between the two intervention and placebo groups over time (from the beginning to the end of week 12). Figure 5 shows the variation in the pain intensity score in the two groups.

## Discussion

DM is the most prevalent disorder of endocrine glands, and many people with DM have a type of the nervous system involvement called DSPN.^39^ In DM, the involvement in peripheral vasculature is high, yet there is no correlation between this type of involvement and diabetic neuropathy; thus, the cause of this disease remains unknown.^40^^-^^44^

Blood glucose control and normalization can relieve the symptoms of neuropathy and prevent the progression of the disease. However, there is evidence that even in case of controlling blood sugar, neuropathy signs are developed.^41^

Over 20% of diabetics suffer from the painful disease of DSPN. It affects the general health level and quality of life even after receiving prescription drugs, including antiepileptic drugs, opioids, and non-steroidal anti-inflammatory drugs.^42^ Recent studies have confirmed the effects of BTX-A on the long-lasting reduction of neuropathic pain in diabetics, compared to previous treatments.^43^^,^^44^

The score of the VAS questionnaire obtained by comparing the patient’s pain score at each stage with the next stage showed that there was a significant decrease in pain in the first week after the BTX-A intervention, implying the positive effect of BTX on treating pain of DSPN. This finding was consistent with results of a meta-analysis conducted by Lakhan et al. who examined the effects of BTX-A on the treatment of DSPN pain. The review results of various studies confirmed the reduction in the VAS test score after the injection of that toxin.^45^

In Iran, a study by Ghasemi et al. showed that patients have experienced a drop in pain and the VAS score after receiving BTX-A, which was consistent with the results of the present research.^32^ On the other hand, we studied quality of life and sleep with other aspects of sensory abnormality more than investigated objects in that study.

Yuan et al. performed a study similar to the present one, reporting that VAS scores decreased in weeks 1, 4, 8, and 12 after the intervention.^31^

In the same vein, Ding et al. reported that the administration of BTX-A reduced the VAS score in weeks 4, 8, 12, and 24 after the injection.^46^

The results of the present study showed that the sleep quality in patients with DM improved after the BTX-A injection; this effect was significant in weeks 4 and 8, implying that the medication effect peaked on day 28 after the intervention. The results of the study by Binay Safer et al. implied a direct correlation between treatment with BTX-A and the improved sleep quality in children.^47^ Our study approved this effect in adult patients.

According to the results of the study by Yuan et al., the PSQI score showed a significant difference in week 4. Although the sleep quality score decreased compared to the pre BTX-A injection period in week 8 and this decremental response implied that the sleep quality increased in the subjects, this was insignificant.^31^ The difference between two studies in the results of week 8 could be due to the difference in the toxin dose because 100 units of BTX-A were used in the present study, yet 50 units of it were administered to the patients in the mentioned study. Another difference between the two studies was the number of the participants, where 18 subjects were divided in two intervention and control groups in the study by Yuan et al.,^31^ but the present study was conducted on 32 subjects. 

In addition, the physical and psychological dimensions of quality of life in both groups were assessed using the SF-36 questionnaire in the present study. In the present study, the psychological dimension of the subjects remained fixed at different times, indicating that BTX-A was psychologically ineffective; nevertheless, the individuals’ level of physical capacity in quality of life improved. This result is very important because a major problem of people with diabetic neuropathy is the poor quality of life and the debilitating complications of their body.^47^

In the study by Yuan et al., no significant correlation was found between treatment with BTX-A and the subjects’ quality of life in both psychological and physical aspects. They attributed the lack of correlation to the impact of the quality of life on various factors, implying that the improvement in neuropathic symptoms alone could not improve the patients’ quality of life.^31^

In the same vein, Ding et al. found out that the injection of BTX-A increased the SF-36 score and the patients’ quality of life in weeks 4, 12, and 24 after the injection.^46^

Considering the NPS questionnaire, the scores of dull, cold, sensitive, deep, and surface sensations decreased from week 4 after the BTX-A injection until the last week, which is another issue to be discussed in the present study. In addition, the scores decreased after weeks 4 and 8 after the injection in the fields of hot, unpleasant, and sharp sensations.

The results of the study by Ghasemi et al. indicated that the score of the NPS questionnaire decreased in all sections, except the cold sensation; therefore, this study was consistent with the results of the present research in all sections apart from the cold sensation.^32^ The difference in the result concerning the sensation parameter could be due to the differences in the ways the two studies were conducted, including the number of patients and the time of monitoring the status of patients after the injection. The present study included 32 patients, while the aforementioned one was conducted on 40 patients; in addition, the present study evaluated patients in weeks 4, 8, and 12 after the injection, while the other one evaluated the patients just once after the injection.

In the same way, Ding et al. reported that NPS scores decreased after treatment with BTX-A in weeks 4, 8, 12, and 24 after the injection.^46^

## Conclusion

The present study showed that the administration of BTX-A could help relieve the pain of diabetic patients with polyneuropathy and help them get rid of the constant suffering from anticonvulsants and sedatives. Changing the dosage of BTX-A produced more desirable outcomes than the former studies; hence, using other doses is likely to produce more optimal results in the future.

## Notes:

**How to cite this article:** Salehi H, Moussaei M, Kamiab Z, Vakilian A. The effects of botulinum toxin type A injection on pain symptoms, quality of life, and sleep quality of patients with diabetic neuropathy: A randomized double-blind clinical trial. Iran J Neurol 2019; 18(3): 99-107.
